# Development of Lateral Flow Test-System for the Immunoassay of Dibutyl Phthalate in Natural Waters

**DOI:** 10.3390/bios12111002

**Published:** 2022-11-10

**Authors:** Anna N. Berlina, Maria Y. Ragozina, Nadezhda S. Komova, Kseniya V. Serebrennikova, Anatoly V. Zherdev, Boris B. Dzantiev

**Affiliations:** A. N. Bach Institute of Biochemistry, Research Center of Biotechnology of the Russian Academy of Sciences, 119071 Moscow, Russia

**Keywords:** phthalates, dibutyl phthalate, environment, contaminant, lateral flow immunoassay, natural water, spring water

## Abstract

The use of a large amount of toxic synthetic materials leads to an increase in the pollution of environmental objects. Phthalates are compounds structurally related to esters of phthalic acid that are widely used in the manufacturing of synthetic packaging materials as plasticizers. Their danger is conditioned by leaching into the environment and penetrating into living organisms with negative consequences and effects on various organs and tissues. This work presents the first development of lateral flow immunoassay to detect dibutyl phthalate, one of the most common representatives of the phthalates group. To form a test zone, a hapten–protein conjugate was synthesized, and gold nanoparticles conjugated with antibodies to dibutyl phthalate were used as a detecting conjugate. The work includes the preparation of immunoreagents, selectivity investigation, and the study of the characteristics of the medium providing a reliable optical signal. Under the selected conditions for the analysis, the detection limit was 33.4 ng/mL, and the working range of the determined concentrations was from 42.4 to 1500 ng/mL. Time of the assay—15 min. The developed technique was successfully applied to detect dibutyl phthalate in natural waters with recovery rates from 75 to 115%.

## 1. Introduction

Disubstituted phthalates (phthalic acid esters) are widely used as plasticizers in consumer products–food packaging, bottles for drinking water, containers for storing food products, and raw materials for their manufacturing. A feature of their inclusion to end products is the absence of covalent chemical bonds; therefore phthalates can migrate into the environment–water and food from packages of non-decomposable waste [1]. The biodegradation of disubstituted phthalates leads to the formation of the more toxic effects of phthalates [2,3,4]. Esters of phthalic acid demonstrate embryotoxic action, damaging effects on DNA, toxic effects on nervous and immune systems, and other negative health effects [5,6]. Their destructive influence on the reproductive system is associated with the chemical similarity with estrogens that causes binding with their receptors [7,8,9], as well as with other pathological pathways, including apoptosis [10]. According to the USEPA, priority phthalates for monitoring are dimethyl phthalate (DMP), diethyl phthalate (DEP), di-n-butyl phthalate (DBP), di-n-octyl phthalate (DnOP), butyl benzyl phthalate (BBP), and di-ethyl-hexyl phthalate (DEHP) [11].

Dibutyl phthalate and other phthalates are released from microplastics, which are recognized as significant environmental pollutants along with chemical wastes [12]. An important feature is the more intense release of DBP in the combined presence of salts and fulvic acids–natural components of soil [12]. In addition to these factors, Ye et al. [13] showed that light emission, the small particle size of the plastic, and elevated temperature are also factors contributing to the release of DBP from plastic. With the growth in the manufacturing and the use of plastic products, the need to control DBP content in water and food is increasing.

The values of the reference dose (RfD) for DBP in rats are 0.1 mg/kg-day (no side effects), 125 mg/kg-day (observed side effects), and 600 mg/kg-day (mortality) [7]. The officially controlled maximum allowable concentrations for DBP in drinking water vary by country and range from 0.2 mg/L [14] to 0.45 mg/L [15,16]. The main sources of DBP entry into the body are drinks, grains, and cereal products [17]. At the same time, some products accumulate phthalates from the package, while others accumulate freely circulating phthalates from the environment. Song et al. [18] showed that DBP is the dominant pollutant of natural river waters among all used phthalate esters, and its concentrations exceed the permissible levels of phthalates in water.

Among the methods used for detecting DBP and other phthalic acid esters, instrumental methods such as gas and liquid chromatography dominate [19,20,21]. These methods have undeniable advantages in terms of sensitivity, selectivity, and reproducibility, but require long sample preparation, as well as a trained specialist to work with the special expensive equipment. However, routine monitoring of pollution requires rapid and efficient analytical methods with sufficient sensitivity and reliability. Most often, immunoassay methods are proposed for this purpose, as they are widely used in clinical practice and environmental monitoring [22]. The advantages of immunochemical analytical techniques are specificity, accuracy, the ability to analyze multiple samples simultaneously, and the possibility of their implementation in most laboratories. Such immunochemical techniques as enzyme immunoassay [23,24], immunofluorescence [25], polarization fluorescent immunoassay [26], and electrochemical immunoassay [27] have been developed and applied to detect dibutyl phthalate in natural waters (rivers), tap water, drinking water, and leachate from plastic bottles in drinking water [28,29].

Due to the absence of lateral flow immunoassays for phthalates in earlier developments, the aim of this work was to develop and characterize this simple and rapid analytical technique. Widely presented dibutyl phthalate was the target analyte. The study integrated the estimation of factors influencing the parameters of the lateral flow assay and finding solutions to ensure sensitive and reproducible measurements. To carry out this work, the necessary reagents have been synthesized, and factors affecting the interaction in the immunochromatographic system were studied.

## 2. Materials and Methods

### 2.1. Materials and Components

Di-n-butyl phthalate (DBP), monobutyl phthalate (MBP), diethyl phthalate (DEP), mono- n-octyl phthalate (MnOP), mono-2-octyl phthalate (M2OP), di- n-octyl phthalate (DnOP), mono benzyl phthalate (MBzP), mono cyclohexyl phthalate (McHP), mono methyl phthalate (MMP), Dimethyl phthalate (DMP), Butyl benzyl phthalate (BBzP), diheptyl phthalate (DHP), monobutyl phthalate (MBP), diethylhexyl phthalate (DEHP), diphenyl phthalate (DPhP), were from Sigma-Aldrich (St. Louis, MO, USA). 4-amino-DBP was synthesized and provided by Prof. Suqing Zhao (Guangdong University of Technology, Guangzhou, China) in lyophilized form. Sodium tetraborate was from Sigma-Aldrich (St. Louis, MO, USA).

Monoclonal antibody to DBP was from Fapon (Songshan Lake, Dongguan, China) and had a concentration of 2.4 mg/mL (in 0.1 M PBS, pH 7.2–7.4). Goat anti-mouse IgG polyclonal antibodies were from IMTEK (1 mg/mL in 0.05 M PBS, pH 7.4, Moscow, Russia). Albumin from bovine serum was from Sigma-Aldrich (St. Louis, MO, USA). Gold (III) chloride and sodium citrate were from Fluka (St. Louis, MO, USA). Dimethylformamide, sodium citrate, potassium carbonate, sodium borate, boric acid, Tris, and Tween-20 were from Sigma-Aldrich (St. Louis, MO, USA). Potassium dihydrogen phosphate, sodium chloride, potassium carbonate, potassium hydroxide, and hydrochloric acid were from Chimmed (Moscow, Russia). All salts, powders, and liquids in their pure form were of analytical grade. The preparation of solutions was carried out in accordance with the rules of work in an analytical laboratory.

Milli-Q water with a resistance of no more than 18.6 MΩ·cm at 25 °C was obtained with the use of the Simplicity Water Purification System (Millipore, Bedford, MA, USA). Both water and water solutions were filtered by 0.22 µm filters. Nitrocellulose membranes (CNPC type) for lateral flow assays were from Advanced Microdevices (MDI, Ambala Cantt, India) and from Millipore (Millipore 180 type, Bedford, MA, USA). Absorption pad CFSP223000 and macroporous CFCP203000 glass-fiber membrane were also from Millipore (Bedford, MA, USA).

### 2.2. Methods

#### 2.2.1. Synthesis of Hapten-Protein Conjugates

The procedure was based on the azo coupling reaction, when the primary amino group of the hapten reacted with the carboxyl group of the protein as a carrier [30]. For this purpose, 12.5 µL of 31% hydrochloric acid, 375 µL of H_2_O, and 15 µL of Tween-20 were added at 0 °C to 5.8 mg of amino-dibutyl phthalate in 25 µL dimethylsulfoxide (DMSO), with the followed addition of 500 µL of water containing 1.375 mg of NaNO_2_.

Next, 10 mg of soybean trypsin inhibitor (STI, carrier protein) was dissolved in 5 mL 0.1 M Na-borate buffer, pH 8.3, and added to the mixture. The color of the reaction mixture changed to terracotta. Then, after incubation for 2 h at room temperature, dialysis was performed against 10 mM phosphate buffer, pH 7.4. The concentration of preparation was calculated from the carrier protein, based on the data of the material balance. The resulting conjugate STI-DBP was aliquoted into small volumes of 10–40 µL and stored at −20 °C until use. In this way, each aliquot was defrosted once before use. The molar ratio STI: DBP under the synthesis was 1:40.

#### 2.2.2. Synthesis of Gold Nanoparticles and Their Conjugation with Antibody

Gold nanoparticles (GNPs) were synthesized via protocol from [31]. First, 200 µL of 5% HAuCl_4_ and 93 mL of Milli-Q water were mixed in the glass vessel and maintained at boiling point. Then, 6 mL of 1% sodium citrate solution in water was added to the solution, and the boiling was continued for the next 5 min. After this, 200 µL of 5% HAuCl_4_ was dropped and kept boiling for 15 min. The freshly synthesized nanoparticles were cooled in the same vessel and used for the conjugation with the antibody. Excess of nanoparticles was stored at 4–6 °C.

Mouse IgG antibody against DBP (mAb) was dialyzed against 10 mM Tris-HCl buffer (pH 8.6). Then, freshly prepared GNPs were pre-adjusted to a pH of 8.6 by 0.2 M K_2_CO_3_. To prepare the conjugate, 2 mL of GNPs were dropped into the glass vessel containing 300 µL of diluted mAb with followed stirring (at Shaker IntelliMixer (ELMI, Riga, Latvia)) for incubation within 45 min at room temperature. Then, 30 µL of 10% solution of BSA in Milli-Q water was added to the mixture, and additional incubation followed under stirring for 15 min at room temperature and 8 h overnight at +4 °C. The obtained conjugate was separated from the unbound antibody and excess amount of BSA by centrifugation at 13,000 *g* at +4 °C for 15 min with decanting of the supernatant. Then, 200 µL of 10 mM Tris-HCl buffer with 2 µL of 10% BSA water solution was added. An optical density of the obtained conjugate was established using a Shimadzu UV-2450 spectrophotometer (Shimadzu, Japan).

#### 2.2.3. Characterization of the Obtained Conjugates

UV-Vis Spectroscopy

Absorption spectra of gold nanoparticles-antibody conjugate, as well as hapten–protein conjugate, were registered by Shimadzu UV-2450 spectrophotometer (Shimadzu, Japan). The optical density of the synthesized conjugate of gold nanoparticles at 527 nm (OD_527 nm_) with the antibody was 11.9.

Fourier transform infrared (FTIR)

The STI-DBP conjugate was lyophilized using a freeze dryer (ALPHA 1–2 LDplus, Martin Christ GmbH, Germany) and stored at 4 °C. The characteristic bands in the spectrum of STI-DBP were measured using Fourier-transform infrared spectroscopy (FT-IR) in the 4000–400 cm^–1^ frequency range was conducted using FT/IR-6700 FT-IR Spectrometer (JASCO Corporation, Japan).

Transmission Electron Microscopy (TEM)

First, 7 uL of native GNPs or the mAb−GNPs conjugate diluted to OD = 1.0 was dropped onto the surface of 300-mesh grids (Pelco International, Redding, CA, USA) with preliminarily formed polyform films. JEM CX-100 electron microscope (JEOL, Tokyo, Japan) was used to obtain images of the nanoparticles, CanoScan 9000F Mark 2 (Canon, Tokyo, Japan)–for scanning microphotographs at 1200 dpi resolution, and Image Tool software (San Antonio, TX, USA)–for data handling.

#### 2.2.4. Characterization of Antibody by ELISA Technique

ELISA was conducted using 96-well transparent polystyrene microplates (Costar 9018, Corning Costar, NY, USA). For this purpose, 100 µL of STI−DBP solution with a concentration 2 µg/mL in 50 mM phosphate buffer saline, pH 7.4, were dropped into wells of microplate and incubated at +4 °C overnight. Then, the microplate was washed three times with 50 mM phosphate buffer, pH 7.4, with 0.05% Tween-20 (PBST). A series of antibody dilutions were added to interact with adsorbed STI−DBP conjugate and incubated for 1 h at +37 °C. Then, after the washing step, anti-species antibody (goat anti-mouse IgG) conjugated with horseradish peroxidase (anti-species conjugate, Jackson, USA, working dilution 1:3000) was added and incubated for 1 h at +37 °C. Next, 100 µL of the substrate solution (commercial TMB + H_2_O_2_ solution, Immunotech, Russia) was added, and after 15 min reaction was stopped by the addition of 50 µL 0.1 M H_2_SO_4_. The optical density was measured at 450 nm and plotted using Origin 9.0 software (OriginLab Corporation, MA, USA). The curve was obtained by plotting the relationship between antibody concentration and the optical density at 450 nm. The concentration of antibody corresponding to OD_450_ = 1.0 was chosen for competitive ELISA.

For the competitive assay and the study of selectivity, the same initial stages were provided. After the incubation of STI−DBP and the washing step, different concentrations of DBP or other structure analogues were added in the volume of 50 µL to the wells. After that, 50 µL of antibody solution in PBST was added, and this mixture was incubated for 1 h at +37 °C. Further stages were carried out as described above, namely adding the anti-species conjugate, developing the resulting complexes with a substrate solution, and measuring the optical density. The curve was obtained by plotting the relationship between the optical density at 450 nm and DBP concentration. The dependences of OD_450_ from the antigen concentration were approximated by a four-parametric sigmoidal equation.

Cross-reactivity values were calculated according to the following equation:

CR(%) = IC50(DBP)/IC50(analogue) × 100%

#### 2.2.5. Composition of Lateral Flow Tests

Assembling of Lateral Flow Tests

**Scheme 1:**
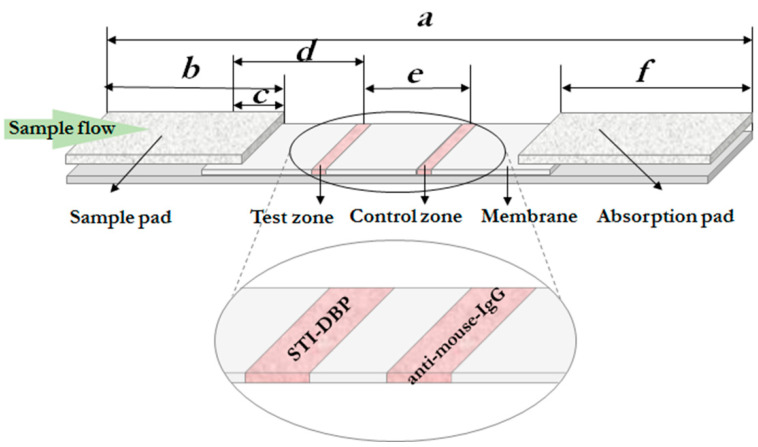
The composition of the test strip used in this investigation. Membrane dimensions and distances: a—60 mm (total length), b—21 mm (sample pad), c—2 mm (overlap), d—12 mm (distance from the beginning of working membrane to test line), e—8 mm (distance between test and control zone), f—16 mm (adsorption pad).

The lateral flow strip was prepared using a working membrane, absorbent membrane (AP 110), and membrane for sample adsorption. STI−DBP and goat-anti-mouse antibodies were immobilized on the working membrane to form the test line and the control line, respectively (Scheme 1). The loading regime used in this work was 0.1 μL per 1 mm. The reagents were dispensed with the use of Isoflow dispenser (Imagene Technology, Inc., Hanover, NH, USA). Then, the multimembrane composite was assembled, dried at 37 °C for 2 h and overnight at 21 ± 1°C, and cut onto the test strips with a width of 3.5 mm with the use of a guillotine cutter (IndexCutter, Port Washington, NY, USA). In the laboratory room used for the manufacturing of test strips, a constant humidity of 27 ± 2% was supported. All dimensional characteristics of the test strip are shown in Scheme 1.

Lateral Flow Assay of DBP

The assay was carried out at room temperature (21 ± 1 °C). First, 100 µL of the probe or 10 mM Tris-HCl buffer solution (pH 8.8) containing 1% Tween-20 with different DBP concentrations were dropped into the microplate wells. After this, 1.0 µL of the conjugate of GNPs with antibody was added to the solution for 10 s. Then, the test strip was inserted vertically by a sample pad for 8 min to provide fluid current over the entire surface of the working membrane. The depth of immersion of the test strip was 5 mm. After that, the test strip was removed and placed horizontally to dry for 5 min.

Collection and Processing Data of Lateral Flow Assays

The color intensity in the test zone after the assay was assessed by processing scanned digital images. The test strips were loaded onto the working surface of the CanoScan 9000F Mark II (Canon, Japan) scanner, and the images were saved and processed. For this purpose, TotalLab software (Nonlinear Dynamics, Newcastle upon Tyne, UK) with 1D regimen was used. When processing the images of the test strips, the image was automatically converted to grayscale, then the test zones were selected in accordance with their location, and the intensity of staining was calculated. Calibration curves were obtained by plotting the relationship between color intensity in the test zone and concentration of DBP in the solution by using Origin 9.0 software (OriginLab) with the use of the four-parametric sigmoidal equation.

#### 2.2.6. Collection and Preparation of Water Samples

The probes of spring water were collected (*n* = 10) from April to July 2022 from points in Russia where springs have existed for more than 20 years. About 50–100 mL of each probe was gathered into the glass vial and sent for storage in the laboratory. For the storage at −20 °C, the probes were aliquoted and frozen. In most cases, water was used without additional pretreatment. If it was needed, spring waters were filtered through a 0.22 µm syringe filter (Millipore, Bedford, MA, USA) to remove insoluble impurities. All the samples were confirmed by gas chromatography as pure from dibutyl phthalate and used for further experiments.

## 3. Results and Discussion

### 3.1. Synthesis and Characterization of DBP–Protein Conjugate

To form a test zone of the lateral flow test, the hapten–protein conjugate was obtained from an amino derivative of DBP and a soybean trypsin inhibitor (STI). As native DBP does not contain a reactive (carboxyl, hydroxyl, or amino) group, its amino derivative substituted in position 4 was used for the synthesis. The conjugation was based on an azo coupling reaction and the use of succinic anhydride [30]. The resulting conjugate was characterized by FT-IR and UV-vis spectrophotometry.

**Figure 1:**
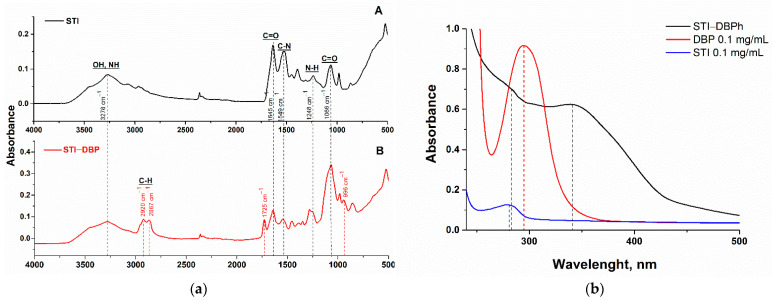
Characterization of STI−DBP by FT-IR and UV−vis spectrophotometric techniques. (**a**) The FT-IR spectra of STI (**black line, A**) and synthesized STI−DBP (**red line, B**); (**b**) UV−vis absorption spectra of STI−DBP conjugate, STI, and DBP.

The FT-IR spectra of the STI−DBP conjugate and the initial STI are shown in Figure 1. In the vibrational spectra of proteins, the amide group of polypeptides can be distinguished by the characteristic frequencies in the regions of 1700–1600 cm^−1^, 1575–1480 cm^−1^, and 1300–1230 cm^−1^ [32]. The peak at 1645 cm^−1^ (Figure 1a) is due to stretching vibrations of the C=O groups [33]. The band at 1549 cm^−1^ is denoted to the C–N stretching vibrations, as well as to the bending of NH in the peak plane [34]. The peak at 1248 cm^−1^ indicates bending vibrations of the N–H bond. The similarity of the absorption peaks is shown in both FT-IR spectra of pure STI (a, black line) and the synthesized STI−DBP (b, red line). Specifically, the band at 1066 cm^−1^ refers to the C=O vibrations. It was recorded both in the spectrum of pure DBP and in the protein molecule (ether bond) with other abovementioned bands. However, after conjugation, the IR spectra revealed the resulting peaks at 1725 cm^−1^ (ester-C=O stretching vibrations) and 996 cm^−1^ (C–O–C stretching vibrations) that are obviously characteristics of dibutyl phthalate, which corresponds to the data on dibutyl phthalate described earlier in the literature [35,36]. The FT-IR spectrum showed the characteristic absorption frequency at 1725 cm^−1^, which is associated with the vibration of the stretching of the carbonyl group of the ester (Figure 1, red line), as well as the absorption peaks at 2920 and 2867 cm^−1^ representing the saturated C–H symmetric and asymmetric stretching vibrations of alkyl groups. The FT-IR spectrum showed the absorption peaks at 2920 and 2867 cm^−1^ representing the saturated C–H symmetric and asymmetric stretching vibrations of alkyl groups of DBP. As a whole, the obtained FT-IR spectra confirmed that the conjugate STI−DBP was successfully synthesized.

The UV-vis absorption spectra of STI, DBP, and the synthesized STI−DBP conjugate are given in Figure 1b. DBP has a strong absorbance peak at 295 nm. The carrier protein STI has a peak at 280 nm. The conjugate shows a peak at 281 nm, which is characteristic of the carrier protein, as well as its own unique absorption in the range of 320–430 nm corresponding to its yellow coloration.

In addition, storage stability was studied up to 1.5 years after synthesis. Since the most labile part of the conjugate is the carrier protein, frequent freezing and thawing can adversely affect its stability [37]. However, when stored in aliquots of up to 50 μL, the necessity of repeated defrosting–freezing cycles was excluded, and the reached retaining activity was confirmed by stable values of LOD and working range for ELISA.

**Figure 2:**
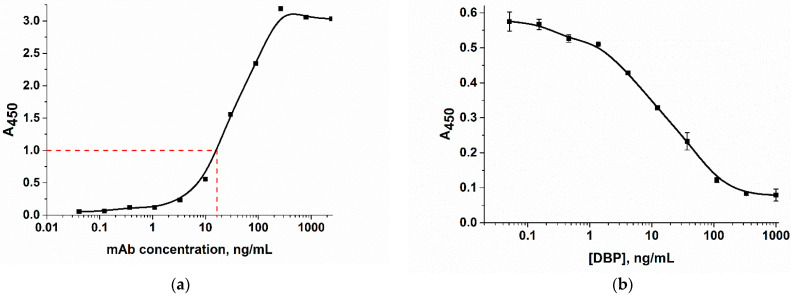
The ELISA curves representing the dependence of registered signal (optical density at 450 nm) from (**a**) antibody concentration, (**b**) DBP concentration (inhibition curve), *n* = 3.

The antibodies were characterized by enzyme immunoassay (Figure 2). Since the oxidized TMB absorbs at 450 nm after adding a stop reagent (1M H_2_SO_4_), all measurements in the ELISA on characterization of the antibodies were carried out with this wavelength. The antibody concentration required to provide the competitive ELISA was determined in non-competitive ELISA. By replacing DBP with other phthalates, the selectivity of antibodies was analyzed. As seen from Figure 2a, the concentration of antibodies corresponding to an optical density of about 1.0 was 16 ng/mL (Figure 2a), and this concentration was chosen for competitive ELISA. Figure 2b shows the competitive ELISA curve obtained for DBP. The detection limit was 0.9 ng/mL and the working range was from 2.5 to 83 ng/mL. The antibodies testing showed their suitability for the development of a lateral flow immunoassay due to the competition at low concentrations of DBP (Figure 2b).

**Figure 3:**
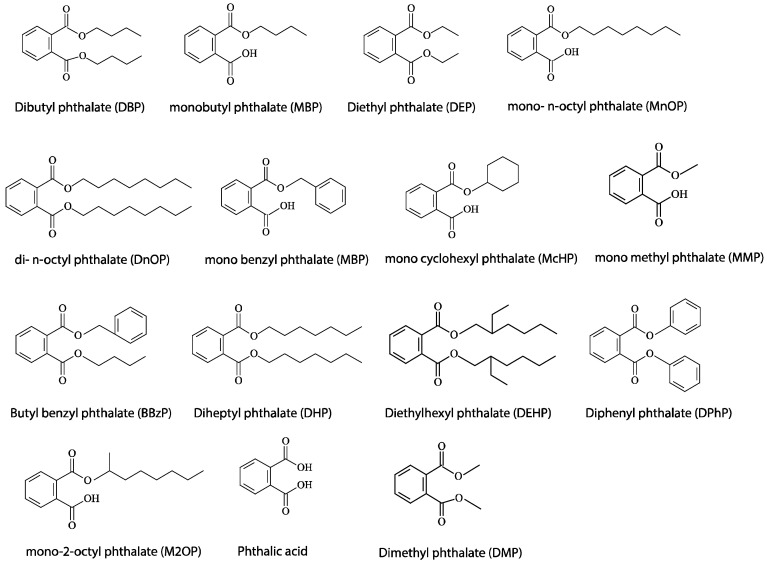
Structural formulas of compounds of the phthalates group used in selectivity testing.

The specificity of the antibody was assessed by ELISA testing of 15 derivatives of phthalic acid esters whose chemical formulas are presented in Figure 3. The compounds were chosen to take into account not only the priority of contamination and production volumes among disubstituted phthalates but also their possible degradation to monoderivatives (Figure 3). The cross-reactivity of the antibody with other structurally related compounds was less than 0.1%.

### 3.2. Synthesis and Characterization of Gold Nanoparticles Conjugated with the Anti-DBP Antibody

Gold nanoparticles are widely used as markers in immunoassays [38,39]. Usually, preparations of GNPs with a diameter of about 30–40 nm are considered as preferable for LFIA [40]. However, the advantages of smaller GNPs for competitive schemes were sometimes noted–13 nm [41,42], 15 nm [43], and 20 nm [44]. The use of larger GNPs in LFIA could be associated with unproductive competition, as described in [45]. For our work, preparations of GNPs with a diameter of about 12 nm were obtained. Characterization of GNPs and their conjugate with antibodies (mAb−GNPs) was provided separately in comparison to each other.

The mAb–GNPs conjugate has been synthesized by physical adsorption. The quantity of the antibodies used for the conjugation was 14 µg per 1 mL of the GNPs’ sol. This amount was calculated to reach complete stabilization of the GNPs and exclude less stable multilayer adsorption. As well as the commonly used 50 mM phosphate buffer, pH 7.4, with 0.1 M NaCl [46] was found to be unacceptable to exclude aggregation, it was replaced with 10 mM Tris-HCl, pH 8.8. The used stabilizing agent was BSA, because its effectiveness has been shown in a number of works [47,48]. In this media, the mAb−GNPs conjugate was stored for 10 months without changes in the properties of its colloid solution.

**Figure 4:**
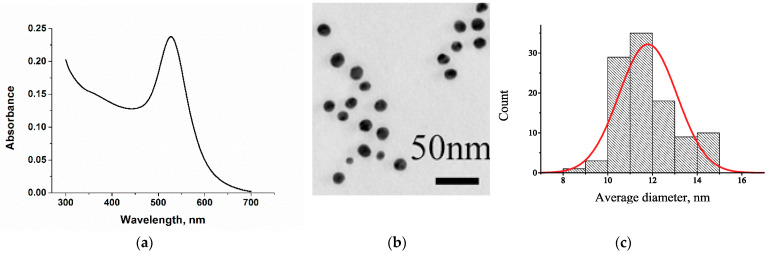
Characterization of the GNP−antibody conjugate. (**a**) Absorption spectrum; (**b**) Fragment of the TEM micrograph; (**c**) histogram showing diameter distribution of the conjugated particles.

According to spectrophotometry, the conjugate had an absorption peak at 527 nm (Figure 4a), which was close to the peak location for the initial GNPs (525 nm). The optical density of the prepared conjugate at the peak wavelength was 11.9. Based on the registered UV-Vis absorption spectrum, the location of their maximums and OD values at these wavelengths were determined. The given parameters were used to control the stability of colloidal solutions of mAb−GNP conjugates [49,50].

A histogram of the distribution of the conjugated particles by size was obtained using TEM data (Figure 4b,c). The average diameter was 11.8 ± 1.3 nm (*n* = 105, minimum value–8.76 nm, maximum value–14.86 nm), with a degree of ellipticity of 1.17 ± 0.11. The absence of aggregates in the preparation and small deviations from the average diameter indicates the receipt of a high-quality preparation.

### 3.3. The LFIA Format

The mAb–GNPs conjugate was incubated with DBP-containing samples for about 10 s for mixing. Then, the test strips were dipped by the sample pad to absorb the mixture. The conjugate moved along the membrane with the liquid flow, and in the absence of DBP in the sample it was bound by free antibody binding sites in the test zone with the formation of a colored band. As the concentration of DBP in the sample increased, the coloration intensity decreased and then disappeared. The sample was completely absorbed and passed the entire distance to the end of the working membrane in less than 8 min, around 2.5 min. We chose a time of 8 min for sufficient accumulation of the analytical signal.

Similarly to the organic hydrophobic dyes studied in our previous work [46], the competition was absent when the conjugate was loaded onto the conjugate pad. This effect probably is caused by the different speeds of analyte and conjugate movement limiting possibilities of their interaction during the lateral flow. Therefore, the pre-incubated LFIA format was applied in the work. The obtained test strips were processed as described in Section 2.2.5 with image conversion to grayscale, but there are other ways to process data [51,52].

### 3.4. The Choice of STI−DBP Immobilization Medium

**Figure 5:**
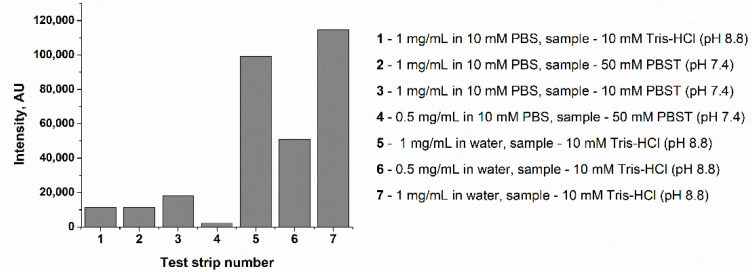
Histogram showing the intensity of signals in the test zone depending on media used for the hapten–protein immobilization (STI−DBP) and sample dilution. OD_527nm_ mAb−GNPs = 0.5. Concentration and medium composition are shown on the right part of the picture.

In the primary experiment, the medium for immobilization of the STI−DBP conjugate was 10 mM PBS, pH 7.4. However, in this case, the binding of the mAb−GNPs conjugate in the test zone was weak (Figure 5, columns 1 and 2). Similarly, weak binding was observed after the addition of 0.05% Tween-20 to the PBS (Figure 5, columns 3 and 4); thus, we have replaced the immobilization medium with Milli-Q. Test strips prepared in this way showed a much more intense binding (Figure 5, columns 5–7).

Changing the medium for the interaction of DBP-containing sample and antibody−GNP conjugate to 10 mM Tris-HCl, pH 8.8, with 1% Tween-20 demonstrated a logical increase in coloration intensity in the test zone (Table 1). Other media were also tested–10 mM borate buffer, pH 9.0, and 10 mM carbonate buffer, pH 10.0, with 1% Tween-20 for better conjugate mobility. The comparison of test zone coloration made it possible to choose the optimal medium–10 mM borate buffer, pH 9.0, see Table 1, strip 2. A similar coloration of the test zone was obtained for measurements in natural spring water (Table 1, strip 4). However, if spring water is diluted with the 10 mM Tris-HCl buffer, pH 8.8, the signal drops (Table 1, strip 3).

### 3.5. Selection of the Working Membrane

The choice of the working membrane in LFIA depends on the medium viscosity and the size of the conjugate particles. The CNPC15 membrane is often used for analysis in various media. Previously, we applied this membrane for LFIA of low molecular weight analytes–antibiotic chloramphenicol and Sudan I dye [53,54], and heavy metals such as lead [55]. However, in the case of DBP, a weak coloration was formed during the interaction in LFIA and the band in the test zone looked pale (Figure 5, columns 1 and 2). A similar situation was observed when working with two hydrophobic compounds, Sudan I [46] and aflatoxin B1 [56]. Due to this, we have changed the working membrane to Millipore 180 (capillary flow rate 180 s/4 cm) which allowed working with water samples (Table 1) unlike the CNPC15 membrane (capillary flow rate is close to 240 s/4 cm).

The choice of membrane for sample absorption also plays an important role. Two sample pads were tested–glass fiber from Millipore non-treated by detergents and pre-treated cellulose PTR7 (MDI). As seen from Table 2, the use of a pre-treated membrane reduces signal intensity in the test zone. Strip 3 showed only 68% coloration intensity in the test zone as compared with strip 1, and strip 4 showed 47% of the value for strip 2. Therefore, a glass fiber membrane was chosen for the sample. In each case, the choice depended on the test zone coloration. The main conditions for choosing the concentrations of the gold conjugate were the presence of an intense signal (at least 20,000 AU) in the absence of DBP and its decrease with increasing analyte concentration (effective competition). The optimization of the analysis conditions is summarized in Table 3.

### 3.6. Analytical Characteristics in Optimized Conditions

**Figure 6:**
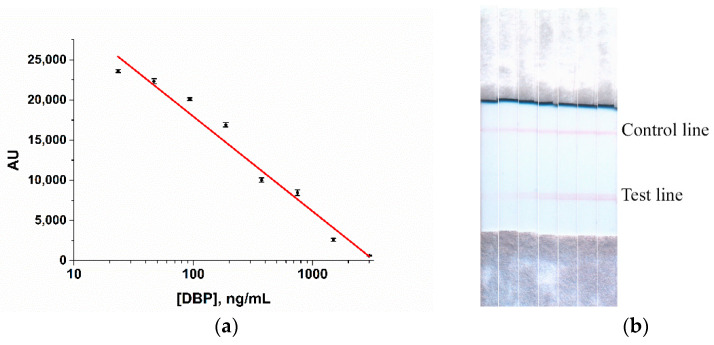
LFIA of DBP in the buffer. (**a**) The linear range of the calibration curve. R2 = 0.984 (*n* = 3), (**b**) The appearance of the test strips when analyzing samples with different content of DBP. Concentrations of DBP from right to the left were 0, 47, 94, 188, 375, 750, 1500 ng/mL.

Under optimized conditions (Table 3), a calibration curve has been obtained, shown in Figure 6. Two detection options are possible–visual (by the disappearance of a band in the test zone in the presence of DBP) and instrumental, based on registered coloration intensities. The cut-off level for visual detection was 1500 ng/mL when the band disappeared; the negative result is when the intensity of staining was less than 2700 arbitrary units. This level has been established as a relative cut-off of visual detection when the test line is not seen by the naked eye. For instrumental registration of the assay results, the calculated limit of detection (LOD) was 33.4 ng/mL, the limit of quantification (LOQ) was 42.4 ng/mL, and the working range was 42.4—1500 ng/mL with linear approximation in semi-logarithmic coordinates. The coefficient of variation was less than 12%. The value of the coefficient of variation in experiments during the day did not exceed 11.5%, and in day-to-day experiments it was no more than 12.8%.

### 3.7. Analysis of Natural Water Samples

The concentration of dibutyl phthalate as well as other phthalic acid esters in natural waters varies depending on the geographical location in a different concentration range–from several ng/mL [57] to several μg/mL [58]. Thus, to determine low concentrations of DBP in natural water, the use of a calibration curve as well as instrumental processing of the data is required. In addition, the analysis of samples with relatively low content of DBP is preferably provided without their dilution to keep the sensitivity and to avoid false-negative results.

**Figure 7:**
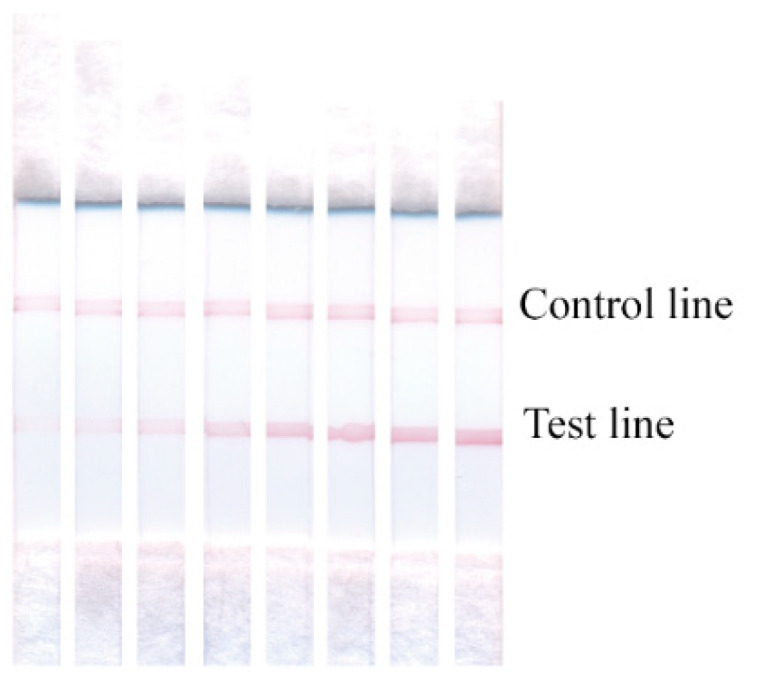
The appearance of the test strips when analyzing spring water with added DBP. Concentrations of DBP from right to the left were 0, 16, 47, 94, 188, 375, 750, and 1500 ng/mL.

The test strips prepared by the chosen optimal protocol were applied to detect DBP in spring water (Figure 7). As mentioned above, the dilution of natural water with buffer decreases coloration in the test zone (Table 1, strip 3). Therefore, water samples were used without dilution. Analysis of spring water samples showed good reproducibility and high recovery of DBP in added-found experiments (Table 4), which demonstrated the absence of the influence of the matrix.

Table S1 integrates data about the possibilities and limitations of different immunoassay formats that were realized for DBP detection (Supplementary Materials). As can be seen, instrumental techniques for testing in laboratory conditions dominate among these developments. Often, they demonstrated low detection limits, but these improvements accord to the concentrations being much lower than MRLs for phthalates. The main trend of the last few years has been to replace the traditionally used peroxidase as a label in ELISA with new markers [57,59,60,61,62]. The proposed LFIA fills an empty niche of simple on-site tests, meeting practical requirements for sensitivity.

## 4. Conclusions

A simple and rapid assay of dibutyl phthalate in natural waters has been developed. Under chosen conditions the developed LFIA technique allowed for the LOD of 33.4 ng/mL. The factors influencing the coloration intensity of the test zone have been established, and the conditions for reliable determination of dibutyl phthalate in spring water samples have been selected. The advantages of the developed test system are rapidity, simplicity, and the possibility to visually detect the presence and evaluate instrumentally the content of dibutyl phthalate in natural waters. The effectiveness of the development has been demonstrated in “added-found” experiments. The obtained results are promising background for dibutyl phthalate assays in other samples. This development solves specific problems associated with the demand for monitoring phthalates in water samples. On the other hand, it is not limited to the target analyte, but considers issues that arise when carefully choosing the conditions of immunochromatographic analysis. It has been shown that varying the environment of immobilization and the interaction medium can significantly improve the characteristics of the system. The article, in our opinion, systematizes approaches to overcome the “underwater rocks” under the development of immunochromatographic test-systems.

## Figures and Tables

**Table 1:** The appearance of the test strips obtained as a result of the selection of running buffer.

No.	Running Buffer	The Appearance of Test Strips	
		Control Line	Test Line
**1**	10 mM Tris-HCl (pH 8.8)	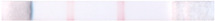	
**2**	10 mM borate buffer (pH 9.0)	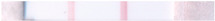	
**3**	spring water/10 mM Tris-HCl (pH 8.8) 1:1	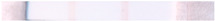	
**4**	spring water	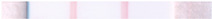	
**5**	10 mM carbonate buffer (pH 9.0)	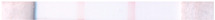	

**Table 2:** The appearance of the test strips obtained as a result of the selection of sample pads.

No.	Conditions, Sample Pad	The Appearance of Test Strip	
		Control Line	Test Line
**1**	10 mM borate buffer (pH 9.0), (OD527 nm mAb−GNPs = 0.06), glass-fiber	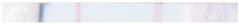	
**2**	10 mM borate buffer (pH 9.0) OD527 nm mAb−GNPs = 0.12, glass-fiber	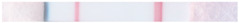	
**3**	10 mM borate buffer (pH 9.0), OD_527_ = 0.06, R7 sample pad	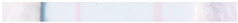	
**4**	10 mM borate buffer (pH 9.0), OD527 Ab−GNPs = 0.12, R7 sample pad	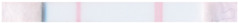	

**Table 3:** Conditions for test strips preparation and LFIA of DBP chosen by sequential optimization.

Parameter	Variations	Signal Intensity, AU	Chosen Conditions
Concentration of GNPs−mAb	OD_527_ = 0.06	10,025	OD_527_ = 0.12
	OD_527_ = 0.12	25,163	
	OD_527_ = 0.24	42,618	
	OD_527_ = 0.5	90,247	
Concentration of STI−DBP	0.25 mg/mL	13,247	0.5 mg/mL
	0.5 mg/mL	25,163	
	1.0 mg/mL	46,319	
	2.0 mg/mL	94,567	
Media for STI−DBP immobilization	10 mM PBS, pH 7.4	4685	Deionized water
	Deionized water	25,163	
Working membrane	CNPC 15	8630	Millipore 180
	Millipore 180	23,645	
Sample pad	Glass-fiber	42,618	Glass-fiber
	PTR7	17,255	
Diluting medium	10 mM borate buffer, pH 9.0	41,845	10 mM borate buffer, pH 9.0
	10 mM carbonate buffer, pH 10.0	17,020	
	10 mM Tris-HCl buffer, pH 8.8	20,784	
	10 mM PBS, pH 7.4	10,940	
Conditions for GAMI immobilization	0.25 mg/mL 10 mM PBS, pH 7.4	25,537	Accords to earlier data

**Table 4:** Determination of DBP in spring water samples by the developed lateral flow assay.

Added DBP	Found DBP/Recoveries					
	Water 1		Water 2		Water 3	
ng/mL	ng/mL	%	ng/mL	%	ng/mL	%
50	43.7	87.4 ± 4.9	43.35	86.7 ± 5.8	39.4	78.5 ± 7.2
100	75.0	75.0 ± 5.9	110.4	110.4 ± 7.4	80.7	80.7 ± 11.2
200	230	115.0 ± 6.5	186.4	93.2 ± 9.4	230	115.0 ± 13.5

## Data Availability

Data are contained within the article. Initial data of instrumental measurements are available on request from the corresponding author.

## Supplementary Materials

The following supporting information can be downloaded at: https://www.mdpi.com/article/10.3390/bios12111002/s1, Table S1: Examples of DBP immunoassays in different samples.
