# Supplementary material for: Development of Lateral Flow Test-System for the Immunoassay of Dibutyl Phthalate in Natural Waters
Source: Biosensors (Basel). 2022 Nov 10;12(11):1002. doi: 10.3390/bios12111002 (PMC9688391; doi:10.3390/bios12111002)
Supplement: Supplementary file 1 [file biosensors-12-01002-s001.zip › biosensors-1986019-supplementary.pdf]

Supplement Information

Development of Lateral Flow Test-System for the Immunoassay of Dibutyl Phthalate in Natural Waters

Anna N. Berlina *, Maria Y. Ragozina, Nadezhda S. Komova, Kseniya V. Serebrennikova, Anatoly V. Zherdev and Boris B. Dzantiev

A. N. Bach Institute of Biochemistry, Research Center of Biotechnology of the Russian Academy of Sciences, 119071 Moscow, Russia
* Correspondence: anberlina@yandex.ru

Table S1. Examples of DBP immunoassays in different samples

№	Type of immunoassay	Analyte	LOD	Working range	Duration of analysis	Sample	Ref.
1	ELISA*	DBP	10.5 ng/mL	25.2–445 ng/mL	3 hours + overnight incubation	tap, lake water	[1]
2	ELISA*	7 PAEs	0.012–0.042 ng/mL	ND	3.5 hours + overnight incubation	greenhouse soils	[2]
3	ELISA*	DBP	21.5 ng/mL	ND	3 hours + overnight incubation	liquor simulants	[3]
4	ELISA*	DBP	4.017 ng/mL	ND	2.3 3 hours + overnight incubation	pure, mineral water, milk, beverages	[4]
5	Dual-signal ELISA*	DBP	0.243 ng/mL<br>0.692 ng/mL	1.00–145 ng/mL<br>1.31–93.1 ng/mL	2.8 hours + overnight incubation	water and beverage	[5]
6	Electrochemica l immmmunoas say*	DBP	0.486 pg/mL	1 pg/mL~1 µg/mL	5.6 hours + double drying at room temperature (no time given)	liquor samples	[6]
7	Dual-label time-resolved fluoroimmuno assay*	DEP<br>DBP	4.9 ng/mL<br>3.9 ng/mL	7.6–103.7 ng/mL<br>6.7–167.2 ng/mL	2.7 hours + overnight incubation	water	[7]
8	Ratiometric fluorescent immunoassay*	DBP	0.86 ng/mL	2.31–66.84 ng/mL	3 hours + overnight incubation	pure water, pond, tap, river water samples	[8]

9	FPIA* or **	DBP	0.35 μg/mL	0.5–7.5 μg/mL	2 min	drinking water from plastic bottles	[9]
10	Immunochromatography** or *	DBP	33.4 ng/mL	42.4 – 1500 ng/mL	15 min	spring water samples	This work

*Laboratory technique
**On-site technique

1. Wei, C.; Ding, S.; You, H.; Zhang, Y.; Wang, Y.; Yang, X.; Yuan, J. An Immunoassay for Dibutyl Phthalate Based on Direct Hapten Linkage to the Polystyrene Surface of Microtiter Plates. *PLOS ONE* **2011**, *6*, e29196. DOI: 10.1371/journal.pone.0029196.
2. Tang, M.; Wei, J.; Du, H.; Zhang, J.; Yang, D.; Peng, Y. Synthesis of an artificial antigen and preparation of a polyclonal antibody for the sensitive determination of phthalate esters by enzyme-linked immunoassay. *Analytical Methods* **2015**, *7*, 3402-3410. DOI: 10.1039/c5ay00079c.
3. Kuang, H.; Liu, L.; Xu, L.; Ma, W.; Guo, L.; Wang, L.; Xu, C. Development of an Enzyme-Linked Immunosorbent Assay for Dibutyl Phthalate in Liquor. *Sensors* **2013**, *13*, 8331-8339.
4. Zhang, Z.; Zhu, N.; Zou, Y.; Wu, X.; Qu, G.; Shi, J. A novel, enzyme-linked immunosorbent assay based on the catalysis of AuNCs@BSA-induced signal amplification for the detection of dibutyl phthalate. *Talanta* **2018**, *179*, 64-69. DOI: 10.1016/j.talanta.2017.10.051.
5. Xiong, D.; Zhu, N.; Zhu, F.; Yakubu, S.; Lv, J.; Liu, J.; Zhang, Z. Investigation and risk assessment of dibutyl phthalate in a typical region by a high-throughput dual-signal immunoassay. *J Hazard Mater* **2022**, *425*, 127991. DOI: 10.1016/j.jhazmat.2021.127991.
6. Li, J.; Jin, H.; Wei, M.; Ren, W.; Wang, J.; Zhang, Y.; Wu, L.; He, B. Dual mode competitive electrochemical immunoassay for dibutyl phthalate detection based on PEI functionalized nitrogen doped graphene-CoSe2/gold nanowires and thionine-Au@Pt core-shell. *Sensors and Actuators B: Chemical* **2021**, *331*, 129401. DOI: 10.1016/j.snb.2020.129401.
7. Zhu, F.; Zhang, H.; Qiu, M.; Wu, N.; Zeng, K.; Du, D. Dual-label time-resolved fluoroimmunoassay as an advantageous approach for investigation of diethyl phthalate & dibutyl phthalate in surface water. *Science of The Total Environment* **2019**, *695*, 133793. DOI: 10.1016/j.scitotenv.2019.133793.
8. Meng, H.; Yao, N.; Zeng, K.; Zhu, N.; Wang, Y.; Zhao, B.; Zhang, Z. A Novel Enzyme-Free Ratiometric Fluorescence Immunoassay Based on Silver Nanoparticles for the Detection of Dibutyl Phthalate from Environmental Waters. *Biosensors* **2022**, *12*, 125.
9. Baranovskaya, V.; Berlina, A.; Eremin, S. A Fluorescence Polarization Immunoassay Procedure for Determining Dibutyl Phthalate in Water. *Journal of Analytical Chemistry* **2022**, *77*, 466-472.
